# Absolute Receptor Occupancy, Not Rate of Decline, Predicts Relapse

**DOI:** 10.1093/schbul/sbag057

**Published:** 2026-06-04

**Authors:** Robert A McCutcheon, Toby Pillinger, Jose M Rubio, David Taylor, Sameer Jauhar

**Affiliations:** Department of Psychiatry, University of Oxford, Oxford OX3 7JX, United Kingdom; TUNE-UP Service, Oxford Health NHS Foundation Trust, Oxford OX3 7JX, United Kingdom; Department of Psychosis Studies, Institute of Psychiatry, Psychology and Neuroscience, King’s College, London SE5 8AF, United Kingdom; Department of Psychiatry, University of Oxford, Oxford OX3 7JX, United Kingdom; TUNE-UP Service, Oxford Health NHS Foundation Trust, Oxford OX3 7JX, United Kingdom; South London & Maudsley NHS Foundation Trust, London SE5 8AF, United Kingdom; Institute of Behavioral Science, Feinstein Institutes for Medical Research, Northwell Health, Glen Oaks, NY 11030, United States; South London & Maudsley NHS Foundation Trust, London SE5 8AF, United Kingdom; Department of Psychiatry, Imperial College London, W12 0NN, London, United Kingdom

**Keywords:** relapse, psychosis, antipsychotic, schizophrenia, clinical trials, discontinuation

## Abstract

Relapse following antipsychotic discontinuation is consistently associated with reduction in dopamine D_2_ receptor occupancy, but available evidence does not support the view that relapse risk is determined by the speed of receptor-occupancy decline. Meta-analytic findings indicate that, within discontinuation studies, abrupt and gradual discontinuation do not significantly differ in relative relapse risk. Apparent advantages of slower dose reduction in some studies are difficult to interpret, as they often reflect continued therapeutic exposure rather than a specific protective effect of tapering itself. Similarly, the non-linear relationship between antipsychotic dose, receptor occupancy, and relapse risk is consistent with a threshold model of therapeutic protection, but does not provide evidence that hyperbolic tapering schedules independently reduce relapse risk. Comparisons of relapse rates across oral and long-acting injectable discontinuation arms from different trials are also methodologically problematic, because substantial trial-level differences affect both discontinuation and maintenance groups. Recent analyses further show that early rapid relapse occurs not only after discontinuation but also in patients maintained on treatment, and that its clinical profile does not resemble a stereotyped withdrawal syndrome. Taken together, the available trial data support the interpretation that relapse risk after discontinuation is primarily determined by whether antipsychotic exposure falls below a therapeutic threshold, rather than by withdrawal kinetics. Although gradual discontinuation may offer pragmatic clinical advantages, there is currently no robust evidence that tapering schedule independently reduces relapse risk.

We thank Dr Horowitz for his interest in our work and the opportunity to further clarify both clinical trial methods and implications of our findings.

In our original study,^1^ we demonstrated that relapse following antipsychotic discontinuation is associated with reduced D_2_ receptor occupancy, but is not related to the speed at which receptor occupancy declines. Our finding is consistent with existing meta-analytic literature,^2^ has since been replicated in a prospective clinical trial,^3^ and is consistent with Dr Horowitz’s own work showing that careful tapering does not reduce relapse risk.^4^ More recently, we have further shown that the early, rapid rate of relapse seen at the start of antipsychotic relapse-prevention studies does not have the profile of a withdrawal phenomenon.^5^

Below, we address the main points raised by Dr Horowitz and explain why, in our view, they reflect a misunderstanding of the literature and trial methodology.

## Evidence from the Clinical Trial Literature

Dr Horowitz incorrectly cites the data from the meta-analysis.^2^ The meta-analysis explicitly states that, in discontinuation studies, abrupt versus gradual discontinuation did *not* significantly affect relative risk of relapse. The paper indicates that abrupt and gradual discontinuation have very similar risks of relapse when compared to continued treatment.

In his letter, Dr Horowitz writes that “abrupt stopping [is] 2.42 times as likely to cause relapse as the slowest tapering.” This is not correct when looking at discontinuation studies abrupt discontinuation (relative risk 2.32) was found to be *less* likely than gradual discontinuation (relative risk 3.27) to cause relapse although this difference was not statistically significant (*P* = .093).

We expect that Dr Horowitz may have instead been examining the results for dose *reduction* rather than discontinuation studies where a relative risk for relapse of 1.02 is reported for reductions taking place over 10 weeks, compared to a relative risk of 2.42 for abrupt reduction. It should be noted that this is not directly relevant to our original findings, and furthermore the gradual dose reduction groups did not have a meaningful dose reduction—ending the studies on average doses of over 5 mg haloperidol equivalents ie, still well within therapeutic occupancy range.

This speaks to a general issue within the field whereby slower tapers inevitably increase the total duration of therapeutic exposure, often leaving patients on higher final doses at any given timepoint, and tend to be used in populations judged to be more stable and motivated. All of these factors would be expected to reduce relapse risk, independent of a specific withdrawal-based mechanism.

Taken together, the meta-analytic literature and more recent discontinuation trials are entirely consistent with our finding that absolute receptor occupancy (ie, remaining above a therapeutic threshold) is the key determinant of relapse risk. Once that threshold is crossed, risk of relapse increases, and the distinction between “abrupt” and “gradual” discontinuation becomes relatively negligible.

## Dose–Response and “Hyperbolic” Tapering

Dr Horowitz invokes Leucht and colleagues’ dose–response analyses as supporting the need for hyperbolic tapering schedules. We do not believe this follows logically from the data.

Leucht’s analysis shows that as receptor occupancy falls, relapse risk increases. It also shows that this is a non-linear relationship ie, going from 60% to 50% occupancy increases relapse risk to a greater extent than going from 90% to 80% occupancy. It does not speak at all to the rate of dose reduction and does not employ data from studies that have examined this.

The hyperbolic relationship between dose and relapse risk is exactly what one would expect from a *receptor-mediated process* in which occupancy saturates at higher doses and falls steeply once doses are reduced to a lower range. The curves clearly show that relapse risk increases markedly once one drops below what is effectively a therapeutic threshold. They do not show that hyperbolic reductions per se reduce relapse risk, beyond the fact that such schedules keep individuals at therapeutic occupancy for longer. In other words, the “hyperbolic” shape of the dose–response curve is a property of dopamine receptor pharmacodynamics, not evidence that a hyperbolic taper schedule has any additional protective effect.

If anything, the Leucht meta-analysis contradicts the hyperbolic tapering proposal. Relapse rates at 0.5 mg of risperidone are highly similar to those on placebo, despite this dose being associated with roughly 30% D_2_ receptor occupancy. At this level, relapse risk is already near maximal. If one accepts the figures of the meta-analysis, it is not clear how spending extended periods making very small reductions at doses below 0.5 mg, as proposed by Dr Horowitz, could meaningfully reduce relapse risk.^6^^,^^7^

## Appropriate Comparison Groups in Relapse-Prevention Trials

A central strand of Dr Horowitz’s argument depends on comparing relapse rates in oral and depot discontinuation arms, from different trials. He infers from cross-trial comparisons that oral discontinuation carries intrinsically higher risk than depot discontinuation and then attributes this difference to the speed of withdrawal.

This ignores the very reason randomized controlled trials include comparator arms.

A plethora of trial level factors will have effects on relapse outcome. These include, though are not limited to population characteristics, recruitment, and stabilization procedures, PANSS baseline deflation, adherence, and duration of pre-randomization period. These effects are not small. Single-arm comparisons across trials, which differ systematically on these dimensions, cannot therefore address the comparison of oral and depot formulations.

**Figure 1:**
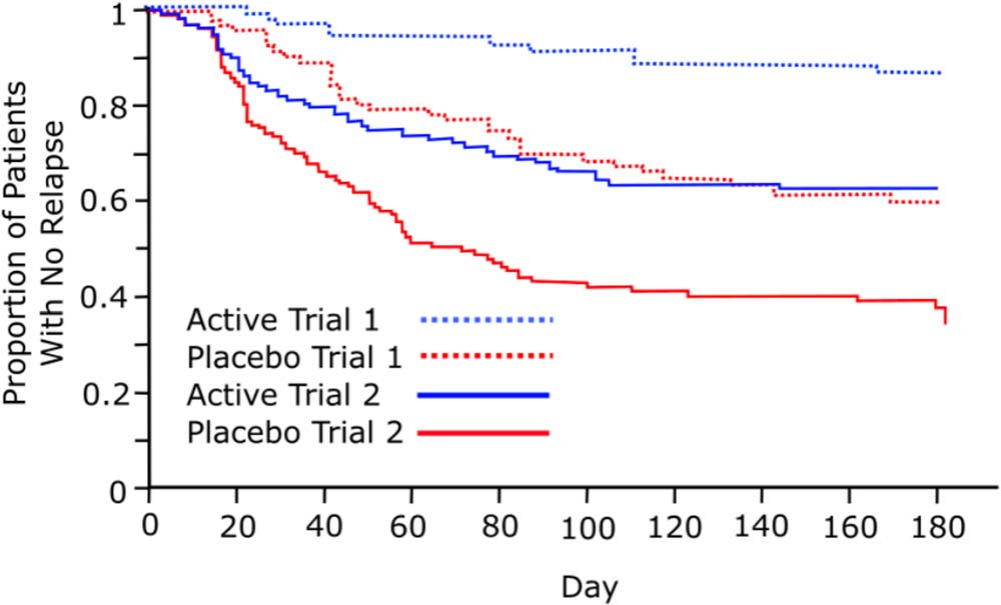
Time from randomization to relapse in two antipsychotic discontinuation trials. Trial 1^10^ is a relapse prevention trial of oral Brexpiprazole (cropped at day 200 for comparability with trial 2). Trial 2 is a relapse prevention trial of oral Aripiprazole.^9^ Despite showing similar hazard ratios between active and discontinuation arms, substantial trial-level effects are apparent, with the initial relapse rate for the continued active-treatment arm in trial 2 being greater than the relapse rate for those abruptly discontinued to placebo in trial 1.

Though this principle should be beyond debate, we further illustrate it with a concrete example. In Figure 1, we show results of two relapse-prevention studies of pharmacologically similar antipsychotics, both using an oral formulation.^8–10^ In both, there is a clear advantage for continuation over discontinuation. However, there are marked differences in relapse rates for both placebo arms. If one were to compare single arms across trials, the placebo arm in trial 1 would, if anything, appear more “protective” than the continuation arm in trial 2.

This is also illustrated in the paliperidone trials considered by both Horowitz and ourselves: oral discontinuation arms show higher relapse rates than depot discontinuation arms, but oral maintenance arms also show higher relapse rates than depot maintenance arms. When the *correct comparison* is made—placebo versus continued treatment within each trial—the apparent advantage for depot discontinuation disappears. It is therefore misleading to attribute the raw differences in discontinuation arms to the speed of withdrawal, while ignoring the equally marked differences in maintenance arms that point to underlying trial design and population effects.

## Clinical Implications and the Meaning of Early Relapse

Dr Horowitz states that our findings imply “it logically follows that abrupt stoppage would be as safe as slow tapering.” This is not a logical consequence of our work and is not a conclusion one can draw from the evidence.

Our paper addressed a more specific mechanistic question: within relapse-prevention trials of paliperidone, is the increased risk of relapse after discontinuation better explained by absolute level of receptor occupancy, or by the rate at which occupancy falls? Our analyses show that the risk of relapse is clearly related to absolute occupancy, and that we find no signal that the rate of occupancy decline adds independent explanatory power in long-acting injectable trials. This does not imply that abrupt discontinuation is generally “as safe” as gradual discontinuation; rather, within the constraints of existing relapse-prevention trials, the dominant pharmacodynamic determinant of relapse is whether occupancy falls below a threshold, not how quickly it does so.

We recently examined this further, analyzing relapse trajectories in both those who relapse following discontinuation and those who relapse while remaining on treatment.^5^ Across multiple relapse-prevention studies, we identified two broad relapse trajectories: an early, rapid relapse and a later, delayed relapse. As expected, relapse overall was more common in discontinuation than continuation groups. However, the proportion of individuals experiencing rapid relapse was almost identical regardless of whether active treatment was continued or discontinued. Moreover, the symptom profile at the time of early relapse did not resemble a stereotypical pharmacological withdrawal syndrome, and there were not significant differences in symptom profile at time of relapse between continuation and discontinuation group.

A plausible explanation for this finding is baseline symptom score deflation at trial enrolment. The requirement to meet eligibility thresholds (typically being relatively symptom-free at randomization) may lead to marginal participants being downrated during baseline screening to allow inclusion. Consistent with this interpretation, in both the long-acting injectable and oral trials, individuals who later experienced rapid relapse had significantly higher symptom severity scores at baseline (time of randomization) than those with delayed relapse. This suggests that symptom severity may have been modestly deflated at entry to fall just below inclusion thresholds. Following randomization, once rating constraints were relaxed, symptom assessments likely reflected true clinical status, giving the appearance of abrupt deterioration. Crucially, this pattern was observed in both discontinuation and continuation arms, which is inconsistent with withdrawal-based hypotheses that would predict rapid relapse specifically following discontinuation.

Taken together with the time-varying hazard analyses in our original paper, these findings argue against the early spike in raw relapse curves in oral discontinuation arms being primarily attributable to pharmacological withdrawal. A more parsimonious explanation is that background trial-level factors—affecting both placebo and continued-treatment arms—contribute to early relapse patterns, as discussed above.

We explicitly state in our paper that gradual discontinuation may have important clinical advantages: it promotes regular clinical contact, allows for more rapid dose reinstatement if symptoms worsen, and helps some patients identify lower effective doses with a better side-effect profile. None of our analyses argue against these pragmatic benefits. Our point is narrower: based on the best available controlled data, there is no robust evidence that the *rate* of receptor-occupancy decline independently determines relapse risk once one accounts for absolute occupancy and trial design.

## Conclusions

There are many narratives one can construct about the mechanism of relapse following antipsychotic discontinuation. The strength of the trial and meta-analytic data is that they allow these narratives to be tested and constrained, as the scientific method demands that hypotheses are evaluated against empirical evidence rather than on intuitive plausibility alone.

Across relapse-prevention trials of paliperidone hazard ratios for discontinuation versus continued treatment are similar for oral and long-acting injectable formulations once one correctly compares within trials. In relapse-prevention trials more broadly, a subset of patients show early “rapid” relapse, but this group is represented in both continuation and discontinuation arms, and their clinical profile does not resemble a withdrawal syndrome. Relapse risk increases during periods in which estimated antipsychotic receptor occupancy falls below a therapeutic threshold, and we find no evidence in long-acting injectable trials that periods of more rapid occupancy decline carry a disproportionate risk once occupancy level is accounted for. Clinical trials have not found gradual discontinuation to reduce relapse rates.^3^^,^^4^ It will be of interest in future to examine large real-world datasets to see if this pattern is also observed in naturalistic settings.

All clinicians share the wish to identify a withdrawal strategy that would allow patients, particularly those who experience significant adverse effects, to discontinue antipsychotics with minimal risk. The evidence to date indicates that discontinuation can be considered in selected patients, but that it inevitably increases risk of relapse, and that there is currently no reliable evidence that any particular withdrawal schedule can eliminate this increased risk, other than by prolonging the period in which patients remain above this protective level of antipsychotic exposure. That hyperbolic dose reduction could have a significant benefit was an intriguing hypothesis that, to date, lacks supporting evidence.

We therefore stand by our original conclusion: in relapse-prevention trials of paliperidone, the increased risk of relapse after discontinuation is best explained by falling below a threshold of dopamine D_2_ receptor occupancy rather than by the speed at which that occupancy declines. The slow and steady accumulation of evidence continues to clarify relapse mechanisms and indicates that absolute occupancy, not withdrawal kinetics, is the primary determinant of risk.
